# Consumption of Fish Products across the Lifespan and Prostate Cancer Risk

**DOI:** 10.1371/journal.pone.0059799

**Published:** 2013-04-17

**Authors:** Johanna E. Torfadottir, Unnur A. Valdimarsdottir, Lorelei A. Mucci, Julie L. Kasperzyk, Katja Fall, Laufey Tryggvadottir, Thor Aspelund, Orn Olafsson, Tamara B. Harris, Eirikur Jonsson, Hrafn Tulinius, Vilmundur Gudnason, Hans-Olov Adami, Meir Stampfer, Laufey Steingrimsdottir

**Affiliations:** 1 Centre of Public Health Sciences, University of Iceland, Reykjavik, Iceland; 2 Educational Research Institute, School of Education, University of Iceland, Reykjavik, Iceland; 3 Department of Epidemiology, Harvard School of Public Health, Boston, Massachusetts, United States of America; 4 Channing Division of Network Medicine, Department of Medicine, Brigham and Women's Hospital and Harvard Medical School, Boston, Massachusetts, United States of America; 5 Örebro University Hospital, Örebro, Sweden; 6 The Icelandic Cancer Registry, Reykjavik, Iceland; 7 Laboratory of Epidemiology, Demography, and Biometry, Intramural Research Program, National Institute on Aging, Bethesda, Maryland, United States of America; 8 Department of Urology, Landspitali – The National University Hospital of Iceland, Reykjavik, Iceland; 9 Department of Medical Epidemiology and Biostatistics, Karolinska Institutet, Stockholm, Sweden; 10 The Icelandic Heart Association, Kópavogur, Iceland; 11 Faculty of Medicine, University of Iceland, Reykjavik, Iceland; 12 Unit for Nutrition Research, Landspitali University Hospital and Faculty of Food Science and Nutrition, School of Health Sciences, University of Iceland, Reykjavik, Iceland; Innsbruck Medical University, Austria

## Abstract

**Objective:**

To examine whether fish and fish oil consumption across the lifespan is associated with a lower risk of prostate cancer.

**Design:**

The study was nested among 2268 men aged 67–96 years in the AGES-Reykjavik cohort study. In 2002 to 2006, dietary habits were assessed, for early life, midlife and later life using a validated food frequency questionnaire. Participants were followed for prostate cancer diagnosis and mortality through 2009 via linkage to nationwide cancer- and mortality registers. Adjusting for potential confounders, we used regression models to estimate odds ratios (ORs) and hazard ratios (HRs) for prostate cancer according to fish and fish oil consumption.

**Results:**

Among the 2268 men, we ascertained 214 prevalent and 133 incident prostate cancer cases, of which 63 had advanced disease. High fish consumption in early- and midlife was not associated with overall or advanced prostate cancer. High intake of salted or smoked fish was associated with a 2-fold increased risk of advanced prostate cancer both in early life (95% CI: 1.08, 3.62) and in later life (95% CI: 1.04, 5.00). Men consuming fish oil in later life had a lower risk of advanced prostate cancer [HR (95%CI): 0.43 (0.19, 0.95)], no association was found for early life or midlife consumption.

**Conclusions:**

Salted or smoked fish may increase risk of advanced prostate cancer, whereas fish oil consumption may be protective against progression of prostate cancer in elderly men. In a setting with very high fish consumption, no association was found between overall fish consumption in early or midlife and prostate cancer risk.

## Introduction

The association between fish consumption – and two important components in certain types of fish, namely long chain n−3 polyunsaturated fatty acids (PUFAs) and vitamin D – and prostate cancer have been investigated in several epidemiologic studies [1]–[5]. Some case-control and cohort studies have reported a reduced risk of prostate cancer and/or prostate-specific mortality by higher fish consumption in adulthood, especially fatty fish consumption [1], [6]–[10], while others have reported the opposite effect, especially for lean fish consumption [6], [11], [12]. Although different source of fish may be of importance, differences in cooking methods may also offer explanations to the mixed findings; cooking white fish using high-temperature has for example been associated with increased risk of advanced prostate cancer [13]. A recent meta-analysis found no association between total fish consumption and overall prostate cancer incidence, but did report a significant reduction in prostate cancer mortality [5]. The analysis did not separately explore different species of fish or method of cooking. Long chain n−3 PUFAs may affect prostate inflammation and carcinogenesis [2] and several studies have reported inverse associations between blood levels of n-3 PUFAs and risk of prostate cancer [14]–[16]. Fatty fish is also a good source of vitamin D and higher prediagnostic plasma 25-hydroxyvitamin D levels have been linked to improved prostate cancer prognosis [17], [18].

Most prior studies assessed diet only from midlife or later. However, we have previously shown the potential role of earlier life diet: a positive association between frequent milk consumption, and an inverse association between frequent rye bread consumption in adolescence and risk of advanced prostate cancer later in life [19], [20]. Only one case-control study of Swedish men diagnosed in the early 1990s, addressed fish intake in early life, and reported a marginally increased risk of prostate cancer later in life [21]. Since the Icelandic population has a tradition of extremely high fish product consumption, we explored consumption of fish, particularly in adolescence and midlife, but also salted or smoked fish and fish oil consumption on prostate cancer risk in the prospective AGES-Reykjavik study.

## Methods

### Ethics Statement

The study protocol was approved by the Icelandic Ethical Review Board (VSNb2007120014/03-7) and the Icelandic Data Protection Authority and written informed consent was obtained from all study participants.

### Study population

The population-based, prospective Reykjavík Study comprises 8894 men aged 33 to 79 years, who resided in the Reykjavik capital area at enrolment (1967–1987). A random sample of 2424 of these men living in 2002 was enrolled in the AGES-Reykjavik study [22].

### Dietary habits in early life, midlife and late life

In the AGES-Reykjavik, 2268 (94%) men – including 214 men with a prostate cancer diagnosis prior to 2002- provided information in year 2002–2006 on dietary habits in early life (between the ages of 14 to 19), midlife (between the ages of 40–50) and current intake (between the ages of 67 to 96) using a food frequency questionnaire (FFQ) [23]. The FFQ assessed frequency of intake of ten common foods and food groups, including fish and fish oil, using the same questions for all three time periods. There were three questions on fish consumption in the FFQ: frequency of fish meals (salted or smoked fish included), fish as topping on bread and in salad, and intake of salted or smoked fish. Response categories for the first two questions were; 1) never, 2) less than once a week, 3) 1–2 times a week, 4) 3–4 times a week, 5) 5–6 times a week 6) daily, and 7) more than once a day. For the salted or smoked fish, response categories were; 1) never, 2) less than once a month, 3) 1–3 times a month, 4) 1–2 times a week, 5) 3–6 times a week, and 6) daily.

Total fish consumption was estimated by converting the weekly average estimates into daily estimates and combining the first two questions, on fish meals and fish as topping on bread and in salad, into one variable. Never became zero fish per day, less than once a week became 0.07 per day, 1–2 times per week became 0.21 per day, 3–4 times a week became 0.5 per day, 5–6 times per week became 0.79 per day, daily became 1 per day and more than once a day became 1.5 per day. The standard portion for a single fish meal was defined as 150 grams and fish on bread as 40 grams, based on average portions in a national nutrition survey [24]. The estimated proportion of fish on bread/salad of a total fish meal was 40/150. The converted numerical value of fish on bread was therefore multiplied by 0.27 and that value computed with the converted value of fish meal per day. The total outcome was then multiplied by 7 to calculate the total consumption per week. Total fish consumption was then divided into three groups i.e. high (>4 portions per week), moderate (>2–4 portions per week) or low (≤2 portions per week).

Although the FFQ had no separate questions for each fish type, cod and haddock were most commonly consumed in the early 20^th^ century [25] and also today [26]. These lean species contain only small amounts of n-3 PUFAs and vitamin D [27]. Fish liver oil supplements in liquid or capsules (hereafter referred to as fish oil), rich in n-3 PUFAs and vitamin D, were evaluated with one question for each period of life, using the same response alternatives as for fish meals, omitting the last option of more than once a day. Cod liver oil is the most common fish oil consumed in Iceland [24] and according to the producer of the fish oil, vitamin D content in 10 milliliters (ml) of cod liver oil, which is the recommended daily dose, was 10 micrograms (400 IU) in the study period. According to the Icelandic food composition database (http://www.matis.is/ISGEM/en/) the amount of eicosapentaenoic acid (EPA) is 0.75 grams (g) in 10 ml of cod liver oil and the amount of docosahexaenoic acid (DHA) is 1.0 g per 10 ml.

Other food groups included in the FFQ were: meat, rye bread, blood sausage or liver sausage, potatoes, fruits, vegetables, milk and milk products and whole wheat bread (not included in the adolescent period).

Our analysis includes men responding to questions on fish and fish oil consumption in early life (ranging from 2257 to 2266 respondents), midlife (ranging from 2257 to 2267 respondents), and current time with prevalent prostate cancer cases excluded (ranging from 2050 to 2055 respondents).

### Validation of the FFQ

The FFQ designed for the AGES-Reykjavik cohort has been validated with regard to midlife and current dietary habits in later life [23], [28]. For current diet a sample of 53 men 65 years or older who answered the AGES-FFQ also filled out a 3-day weighed food record. A significant correlation was found between these two methods of reporting current intake of fish oil (ρ = 0.51, *P*<0.001), but not for fish meals and fish toppings (ρ = 0.23, *P* = 0.098 and ρ = 1.23, *P* = 0.146, respectively) [28]. Because of low validity for overall current fish intake, these data were not used to study prostate cancer risk. However, due to less frequent consumption of salted or smoked fish in later life it was not possible to validate the question in a 3-day weighed food record and therefore not included in the validation study.

For midlife dietary habits, retrospective food consumption of 56–72 year-old participants (n = 67) was estimated by comparing the results in the AGES-FFQ with detailed dietary data (an hour-long interview on dietary habits in the past 3 months) gathered from the same individuals 18–19 years previously. The strongest correlation was found for fish oil (ρ = 0.53, *P*<0.001) while the correlation coefficient for fish consumption was 0.26 (*P* = 0.037) [23].

The validity of the early life dietary assessment has not been investigated. Yet, the data show similar residency-dependent variation in dietary habits as documented in a household study conducted in Iceland in 1939 [25]. Among those who had early life residency in a coastal village 46 percent consumed more than four portions of fish per week in early life, while 35 percent consumed high amount of fish in the capital area and 33 percent in rural area. Fish oil consumption (once a week or more often) in early life was similar (68%) in coastal villages and the capital area, while 57 percent of rural area residents reported fish oil consumption. Lastly, high consumption of salted or smoked fish in early life was most frequent among those who grew up in a rural area i.e. 69 percent and 58 percent in coastal villages and 46 percent in the capital area.

### Covariate Assessment

Information on potential confounders in midlife was retrieved from the questionnaire or health check-ups at entry to the Reykjavik Study and has been described elsewhere [19], [20]. Information on nutritional factors such as fish-, fish liver oil-, meat- and milk intake in all time periods were obtained from the FFQ in the AGES-Reykjavik study as well as recall information about physical activity in the past [22].

### Ascertainment of outcome

We ascertained prostate cancer diagnoses through linkage with the nationwide Icelandic Cancer Register, which captures 99% of cancers diagnosed in Iceland [29]–[31]. Information on cause of death was obtained from Statistics Iceland. Based on medical records, stage at diagnosis was classified as stage I (incidental finding; T1a, NX/0, and MX/0); Stage II (tumor confined to prostate gland; T1b/1c/1/2, NX/0, and MX/0); Stage III (tumor extending through prostatic capsule; included T3, NX/0, and MX/0) and stage IV (locally advanced or metastatic disease; T4, NX/0, MX/0; or any T, N1 and/or M1). We had information on stage for approximately 75% of cases. Information on Gleason grade was not available. Since 1990 prostate cancer incidence rates in Iceland have increased rapidly with no substantial trend in mortality, suggesting increased detection of nonlethal tumors [32].

Men who died from prostate cancer or had stage III or IV at diagnosis were classified as having advanced prostate cancer. We retrieved information on cancer diagnosis (including cancers that were prevalent among participants in AGES-Reykjavik) and mortality through December 31, 2009. For incident cases, participants were followed from study entry into the AGES-Reykjavik (between 2002 and 2006) where they provided the dietary information, until diagnosis of prostate cancer, death or the end of the observation period (December 31, 2009). Because of computerized national roster that includes an individually unique national registration number for each person, follow-up is virtually complete [33]. With three measurements of dietary habits across the lifetime it is clear that the design of the study is not the same throughout. When analyzing the early and midlife dietary patterns, we include both prevalent and incident cases diagnosed in the period from 1981 to 2009. When analyzing the later life exposure, we only include prostate cancer cases who answered the FFQ before being diagnosed (from 2002 to 2009).

### Statistical Analyses

Our analyses of early-life and mid-life diet included both incident and prevalent cancer, and thus we used logistic regression models to calculate odds ratios (ORs) and 95% confidence intervals (CIs). We compared men who were high (>4 portions per week), moderate (>2–4 portions per week) or low (≤2 portions per week) consumers of total fish. For advanced prostate cancer, we collapsed moderate and high intake groups given the small number of cases. Only six participants never consumed fish products in early life and two never in midlife.

Information on potential confounders in midlife was retrieved from the questionnaire or health check-ups at entry to the Reykjavik Study. We adjusted in multivariate models for birth year (continuous), age at study entry in midlife (continuous), height (continuous), BMI (≥30, <30 kg/m^2^), type 2 diabetes in midlife, education (three categories: elementary or secondary school; college education; university education), family history of prostate disease, seeing a physician regularly, fish oil consumption (never vs. once a week or more); rye bread consumption (daily or more vs. less than daily); meat consumption (up to 2/week vs. 3+/week), and milk intake (daily or more vs. less than daily). Smoking habits and physical activity were excluded from the models because they did not affect the risk estimates.

Similarly, in the analysis of fish oil consumption we compared once a week or more with never consumers in all three time periods. For salted or smoked fish we contrasted high (1+/week) with less frequent consumers (≤3/month). Only 54 participants never consumed salted or smoked fish in early life, 82 in midlife and 340 in current time. We also divided salted or smoked fish intake in early life into three groups and explored the association with advanced prostate cancer risk: three times a month or less (reference group, 43.7% of participants), 1–2 times per week (43.6% of participants) and four times per week or more (12.6%). We then employed a trend analysis using these three groups as a continuous variable. In the multivariate analysis we used the same covariates as for the total fish analyses. Lastly, the salted and smoked fish consumption in midlife and early life was pooled in one variable with four categories to assess potential effects of longitudinal consumption on advanced prostate cancer risk.

When we analyzed later life consumption of fish oil and salted or smoked fish (1+ p/month vs. <1 p/month), 214 men with prevalent prostate cancer were excluded, and we used Cox proportional hazard regression models to calculate hazard ratios (HRs) and 95% CIs of total (n = 133) or advanced prostate cancer (n = 27), using the same covariates as in the early- and midlife analyses.

In all statistical analyses, we used SPSS software, version 19.0 (SPSS Inc., 2009, IBM Chicago, IL, www.spss.com).

## Results

### Overall findings

The mean age (±SD) was 46.8±6.9 years when the participants entered the Reykjavik Study and 76.6±5.3 years when they entered the AGES-Reykjavik component and provided the dietary information. Table 1 shows characteristics, mostly collected in midlife in the Reykjavik Study, of the men who reported their dietary habits (n = 2268).

**Table 1:** Characteristics of participants reporting dietary habits in the study.

	All	Salted or smoked fish intake in adolescence		Salted or smoked fish intake in later life		Fish oil intake in later life	
	N = 2268	High^1^, n = 1270	Low^2^, n = 987	High^3^ n = 815	Low^4^, n = 1450	Yes, n = 1719	No, n = 543
Age (y)^5^	76.6±5.3	77.2±5.4	75.7±5.1	77.0±5.4	76.3±5.3	76.5±5.3	76.6±5.6
**Education (%)**							
-Primary and Secondary	1663 (73.3)	933 (73.5)	725 (73.5)	623 (76.4)	1041 (71.8)	1258 (73.2)	401 (73.8)
-College	314 (13.8)	179 (14.1)	132 (13.4)	107 (13.1)	204 (14.1)	232 (13.5)	81 (14.9)
-University	291 (12.8)	158 (12.4)	130 (13.2)	85 (10.4)	205 (14.1)	229 (13.3)	61 (11.2)
**Prostate disease in the family (%)**	224 (9.9)	135 (10.6)	86 (8.7)	78 (9.6)	147 (10.1)	176 (10.2)	49 (9.0)
**Regular health check-up (%)**	421 (18.6)	228 (18.0)	189 (19.1)	126 (15.5)	292 (20.1)	324 (18.8)	95 (17.5)
**Diabetes type 2 in midlife (%)**	38 (1.7)	23 (1.8)	15 (1.5)	18 (2.2)	20 (1.4)	33 (1.9)	5 (0.9)
**Smoking status in midlife (%)**							
-Never	575 (25.4)	323 (25.4)	249 (25.2)	178 (21.8)	395 (27.2)	442 (25.7)	129 (23.8)
-Previously	529 (23.3)	290 (22.8)	237 (24.0)	171 (21.0)	357 (24.6)	415 (24.1)	113 (20.8)
-Current	1164 (51.3)	657 (51.7)	501 (50.8)	466 (57.2)	698 (48.1)	862 (50.1)	301 (55.4)
**Early life residency (%)** 6							
-Reykjavik	816 (36.9)	372 (29.9)	439 (45.8)	248 (31.1)	568 (40.2)	612 (36.5)	202 (38.0)
-Sea village	724 (32.7)	420 (33.8)	303 (31.6)	275 (34.5)	450 (31.8)	555 (32.9)	172 (32.3)
-Rural area	599 (27.1)	413 (33.2)	182 (19.0)	242 (30.4)	354 (25.1)	452 (27.0)	144 (27.1)
-Combination of rural area / sea village	74 (3.3)	38 (3.1)	35 (3.6)	32 (4.0)	41 (2.9)	60 (3.6)	14 (2.6)
**BMI (m/kg2) ≥ 30 in midlife (%)**	163 (7.2)	96 (7.6)	67 (6.8)	66 (8.1)	97 (6.7)	111 (6.5)	52 (9.6)
**BMI (m/kg** 2 **) in midlife** 5	25.5±3.1	25.5±3.1	25.4±3.1	25.4±3.1	25.5±3.1	25.5±3.0	25.5±3.4
**Height (cm) in midlife** 5	177.9±6.0	177.9±6.0	178.2±5.9	177.6±6.1	178.2±6.0	177.9±6.0	177.9±6.0
**Total prostate cancer (%)** 7	247 (15.3)	203 (16.0)	140 (14.2)	47 (6.3)	86 (6.6)	94 (6.0)	39 (7.9)
-Localized	284 (12.5)	158 (12.5)	124 (12.6)	31 (4.2)	75 (5.7)	77 (5.0)	29 (5.8)
-Advanced	63 (2.8)	45 (3.6)	16 (1.6)	16 (2.2)	11 (0.8)	17 (1.1)	10 (2.0)

1 Consuming salted or smoked fish once a week or more often.

2 Consuming salted or smoked fish three times a month or less.

3 Consuming salted or smoked fish once a month or more often.

4 Consuming salted or smoked fish less than once a month.

5 Values are means ± SDs.

6 Data on residency is missing for 55 men.

7 For dietary patterns in later life, only the number of incident prostate cancer cases in the study is shown.

Table 2 shows dietary habits among the participants across three periods of life and reflects the variability in food availability in Iceland across time. The correlation coefficients are generally weak for individual food items between adolescent diet and current diet with the strongest positive correlation for rye bread (ρ = 0.36, *P*<0.001). Meat consumption was the only food item that showed negative correlation (ρ = −0.06, *P* = 0.008), suggesting a shift in consumption between regional areas and/or social groups during this study period. Dietary habits also changed markedly during the study period with high consumption of salted or smoked fish declining from 60% to 7% from 1907 to 2006. The highest category of total fish consumption, over 4 portions per week, also declined from 38% to 21%, whereas daily fish oil consumption increased from 30% to 61%, most probably due to greater access.

**Table 2:** Dietary habits among participants through different time-periods.

	Adolescence	Midlife	Later life	Spearmans's ρ	P
	n (%)	n (%)	n (%)		
**Fish**				0.11	<0.001
≤2 portions p/w	1097 (48.4)	300 (13.2)	596 (26.3)		
>2 up to 4 portions p/w	301 (13.3)	1287 (56.8)	1203 (53.0)		
>4 portions p/w	868 (38.3)	680 (30.0)	470 (20.7)		
**Salted or smoked fish**				0.19	<0.001
3 times a month or less	987 (43.7)	1465 (64.8)	2113 (93.3)		
once p/w or more	1270 (56.3)	797 (35.2)	152 (6.7)		
**Fish oil**				0.19	<0.001
never	800 (35.4)	585 (25.9)	543 (24.0)		
6 times p/w or less	777 (34.2)	689 (30.5)	348 (15.4)		
daily	688 (30.4)	984 (43.6)	1371 (60.6)		
**Milk and milk products**				0.25	<0.001
less than daily	463 (20.4)	834 (36.9)	1066 (47.1)		
daily or more	1804 (79.6)	1425 (63.1)	1197 (52.9)		
**Rye bread**				0.36	<0.001
less than daily	1212 (53.7)	1582 (70.0)	1659 (73.4)		
daily or more	1046 (46.3)	678 (30.0)	601 (26.6)		
**Meat**				−0.06	0.008
2 times p/w or less	864 (38.2)	734 (32.5)	696 (30.7)		
3 times p/w or more	1397 (61.8)	1525 (67.5)	1571 (69.3)		
**Salted or smoked meat**				0.26	<0.001
3 times a month or less	1411 (62.5)	1560 (68.9)	2065 (91.3)		
once p/w or more	847 (37.5)	703 (31.1)	197 (8.7)		
**Fruits**				0.07	0.001
never	820 (36.3)	134 (5.9)	24 (1.1)		
6 times p/w or less	1416 (62.7)	2024 (89.6)	1517 (67.0)		
daily	21 (0.9)	102 (4.5)	724 (32.0)		
**Vegetables**				0.23	0.000
never	563 (24.9)	176 (7.8)	167 (7.4)		
6 times p/w or less	1663 (73.6)	1967 (87.2)	1883 (83.1)		
daily	35 (1.5)	114 (5.1)	217 (9.6)		

Spearman's correlation analysis was performed when comparing dietary habits in adolescence and later life.

*P* values were calculated by using Spearmans's correlation analysis.

Of the 2268 men, 347 had been or were diagnosed with prostate cancer during follow-up, 63 with advanced disease. The mean age (± SD) at cancer diagnosis was 74.8±6.5 years. After completion of the FFQ, 133 men were diagnosed with prostate cancer, of which 27 had advanced disease. When we analyzed current dietary habits assessed at entry to the AGES-Reykjavik study, the 214 prevalent cases were excluded. The mean follow-up time (± SD) from entry to the AGES-Reykjavik until diagnosis of prostate cancer, death or the end of the observation period was 5.1±1.6 years.

### Fish intake

High total fish consumption in early and midlife was not associated with total, or advanced prostate cancer (Table 3). Likewise, high intake of salted or smoked fish during early life and midlife showed no association with total prostate cancer (Table 4). However, men consuming salted or smoked fish once a week or more often during adolescence were at 2-fold increased risk for advanced prostate cancer (95% CI: 1.08, 3.62) compared with consumption 3 times per month or less. When we added early life residency to the multivariate model, the risk estimate increased slightly [OR (95% CI): 2.16 (1.13, 4.12)]. We further divided salted or smoked fish intake into three groups and explored the association with advanced prostate cancer risk. We found a significant trend with risk estimates of 1–2 times per week [OR (95% CI): 1.93 (1.03, 3.60], and three times per week or more [OR (95% CI): 2.18 (0.94, 5.06)] compared with three times a month or less (*P*_trend_  = 0.03).

**Table 3:** Prostate cancer (PCa) risk by total fish consumption in early- and midlife.

	PCa (n)	Age adjusted OR (95% CI)		OR^1^ (95% CI)	*P*_trend_	PCa (n)	Age adjusted OR (95% CI)	OR^1^ (95% CI)	*P*_trend_	PCa (n)	Age adjusted OR (95% CI)	OR^1^ (95% CI)
Adolescence (14–19 years)	*Total n = 344*					*Localized n = 282*				*Advanced n = 62*		
**N = 1984**												
≤2 portions p/w										32	1.0	1.0
>2 portions p/w										30	0.77 (0.46, 1.29)	0.81 (0.46, 1.42)
**N = 2266–2204**												
≤2 portions p/w	173		1.0	1.0	0.245	141	1.0	1.0	0.325			
>2 up to 4 portions p/w	49		1.03 (0.73, 1.46)	1.05 (0.74, 1.51)		41	1.06 (0.73, 1.55)	1.12 (0.76, 1.65)				
>4 portions p/w	122		0.83 (0.65, 1.07)	0.87 (0.66, 1.13)		100	0.85 (0.65, 1.12)	0.86 (0.65, 1.14)				

Midlife (40–50 years)	*Total n = 345*					*Localized n = 282*				*Advanced n = 63*		
**N = 1985**												
≤2 portions p/w										9	1.0	1.0
>2 portions p/w										54	0.81 (0.39, 1.67)	0.74 (0.35, 1.56)
**N = 2267–2204**												
≤2 portions p/w	45	1.0		1.0	0.783	36	1.0	1.0	0.606			
>2 up to 4 portions p/w	189	0.93 (0.65, 1.33)		0.96 (0.66, 1.38)		155	0.93 (0.65, 1.33)	0.96 (0.66, 1.38)				
>4 portions p/w	111	1.02 (0.70, 1.49)		1.05 (0.71, 1.57)		91	1.02 (0.70, 1.49)	1.05 (0.71, 1.57)				

1 Adjustment made for birth year, age at study entry in midlife, education, family history of prostate disease, going to a physician regularly, height in midlife, BMI in midlife, type 2 diabetes in midlife and concurrent salted or smoked fish-, fish oil-, milk-, rye bread-, and meat intake.

**Table 4:** Prostate cancer (PCa) risk by salted or smoked fish consumption across the lifespan.

	PCa (n)	Age adjusted OR (95% CI)	OR^1^ (95% CI)	PCa (n)	Age adjusted OR (95% CI)	OR^1^ (95% CI)	PCa (n)	Age adjusted OR (95% CI)	OR^1^ (95% CI)
Adolescence (14–19 years)	*Total n = 343*			*Localized n = 282*			*Advanced n = 61*		
**N = 2257–1975**									
3 times a month or less	140	1.0	1.0	124	1.0	1.0	16	1.0	1.0
Once a week or more	203	1.08 (0.85, 1.37)	1.09 (0.85, 1.39)	158	0.96 (0.75, 1.24)	0.99(0.76, 1.28)	45	1.96 (1.09, 3.51)	1.98 (1.08, 3.62)

Midlife (40–50 years)	*Total n = 343*			*Localized n = 282*			*Advanced n = 61*		
**N = 2257–1975**									
3 times a month or less	224	1.0	1.0	186	1.0	1.0	38	1.0	1.0
Once a week or more	119	0.91 (0.72, 1.17)	0.96 (0.74, 1.24)	94	0.88 (0.67, 1.15)	0.92 (0.69, 1.21)	25	1.09 (0.65, 1.83)	1.27 (0.74, 2.19)

Later life (67–96 years)	*Total n = 133*	Age adjusted HR (95% CI)	HR^1^ (95% CI)	*Localized n = 106*	Age adjusted HR (95% CI)	HR^1^ (95% CI)	*Advanced n = 27*	Age adjusted HR (95% CI)	HR^1^ (95% CI)
**N = 2054–1948**									
Once a month or less	86	1.0	1.0	75	1.0	1.0	11	1.0	1.0
Once a month or more	47	0.99 (0.69, 1.41)	0.99 (0.69, 1.42)	31	0.78 (0.51, 1.19)	0.63 (0.40, 1.00)	16	2.53 (1.17, 5.45)	2.28 (1.04, 5.00)

1 Adjustment made for birth year, age at study entry in midlife, education, family history of prostate disease, going to a physician regularly, height in midlife, BMI in midlife, type 2 diabetes in midlife and concurrent total fish-, fish oil-, milk-, rye bread-, and meat intake.

High intake of salted or smoked fish in midlife (Table 4) showed no statistically significant association with advanced prostate cancer. However, we found a positive association with high consumption (1+ p/month vs. <1 p/month) of salted or smoked fish in later life [HR (95% CI): 2.28 (1.04, 5.00)] and advanced prostate cancer. When we added early life residency to the fully adjusted model, the risk estimate was minimally affected [HR (95% CI): 2.15 (0.96, 4.81)]. None of those frequent consumers of salted or smoked fish in later life (1+ p/month vs. <1 p/month) with advanced prostate cancer were also frequent consumers in early life.

Table 5 presents ORs and 95% CIs of advanced prostate cancer by salted or smoked fish consumption both in adolescence and midlife. Compared with low consumption in both time periods a positive association was only observed when the consumption was high in both time periods [OR (95% CI): 2.14 (1.05, 4.35)].

**Table 5:** Prostate cancer risk by longitudinal salted or smoked fish consumption.

Advanced prostate cancer (n = 59)			
Adolescence	Midlife	Number	OR^1^ (95%CI)
Low^2^	Low	821	1.00
Low	High^3^	162	0.84 (0.19, 3.79)
High	Low	634	1.75 (0.86, 3.57)
High	High	633	2.14 (1.05, 4.35)

1 Adjustment made for birth year, age at study entry in midlife, education, family history of prostate disease, going to a physician regularly, height in midlife, BMI in midlife, type 2 diabetes in midlife, rye bread-, fish-, fish liver oil-, meat-, and milk intake in adolescence.

2 Three times a month or less.

3 Once a week or more.

### Fish oil

Early or midlife consumption of fish oil was not statistically significant associated with total prostate cancer or with advanced disease (Table 6). For current fish oil consumption in later life no statistically significant association was found with total prostate cancer [HR (95% CI): 0.72 (0.48, 1.06)]. However, those consuming fish oil once a week or more often in later life were at decreased risk for advanced disease compared with those who never consumed fish oil [HR (95% CI): 0.43 (0.19–0.95)]. When we added early life residency to the multivariate model, the risk estimate for advanced disease was similar [HR (95% CI): 0.45 (0.20–1.03)].

**Table 6:** Prostate cancer (PCa) risk by fish oil consumption across the lifespan.

	PCa (n)	Age adjusted OR (95% CI)	OR^1^ (95% CI)	*P*_trend_	PCa (n)	Age adjusted OR (95% CI)	OR^1^ (95% CI)	*P*_trend_	PCa (n)	Age adjusted OR (95% CI)	OR^1^ (95% CI)
Adolescence (14–19 years)	*Total n = 345*				*Localized n = 282*				*Advanced n = 63*		
**N = 1979**											
Never									25	1.0	1.0
Once a week or more									38	0.91 (0.54, 1.52)	0.92 (0.53, 1.60)
**N = 2261–2198**											
Never	119	1.0	1.0	0.695	94	1.0	1.0	0.452			
6 times p/w or less	119	1.09 (0.83, 1.44)	1.10 (0.83, 1.46)		97	1.11 (0.82, 1.51)	1.10 (0.81, 1.51)				
Daily	107	1.07 (0.80, 1.42)	1.06 (0.79, 1.42)		91	1.15 (0.84, 1.56)	1.13 (0.82, 1.54)				

Midlife (40–50 years)	*Total n = 343*				*Localized n = 280*				*Advanced n = 63*		
**N = 1978**											
Never									16	1.0	1.0
Once a week or more									47	1.08 (0.60, 1.92)	1.02 (0.56, 1.85)
**N = 2258–2195**											
Never	95	1.0	1.0	0.563	79	1.0	1.0	0.546			
6 times p/w or less	96	0.87 (0.64, 1.18)	0.82 (0.60, 1.12)		76	0.81 (0.58, 1.14)	0.77 (0.55, 1.09)				
Daily	152	0.95 (0.72, 1.26)	0.90 (0.68, 1.20)		125	0.93 (0.69, 1.26)	0.89 (0.65, 1.21)				

Later life (67-96 years)	*Total n = 133*	Age adjusted HR (95% CI)	HR^1^ (95% CI)		*Localized n = 106*	Age adjusted HR (95% CI)	HR^1^ (95% CI)		*Advanced n = 27*	Age adjusted HR (95% CI)	HR^1^ (95% CI)
**N = 1944**											
Never									10	1.0	1.0
Once a week or more									17	0.52 (0.24, 1.13)	0.43 (0.19, 0.95)
**N = 2050–2023**											
Never	39	1.0	1.0	0.119	29	1.0	1.0	0.874			
6 times p/w or less	20	0.74 (0.43, 1.28)	0.70 (0.40, 1.20)		16	1.06 (0.57, 1.99)	1.27 (0.62, 2.59)				
Daily	74	0.74 (0.50, 1.09)	0.72 (0.48, 1.06)		61	1.31 (0.83, 2.06)	1.06 (0.64, 1.76)				

1 Adjustment made for birth year, age at study entry in midlife, education, family history of prostate disease, going to a physician regularly, height in midlife, BMI in midlife, type 2 diabetes in midlife and concurrent total fish-, salted or smoked fish-, milk-, rye bread-, and meat intake.

## Discussion

In this population-based study of Icelandic men, consumption of total salted or smoked fish and intake of fish oil during different periods of life was not associated with risk of prostate cancer overall. In contrast, the risk of advanced prostate cancer was increased following high intake of smoked or salted fish during adolescence and late life, and substantially reduced among men who consumed fish oil at older age. Although our results for total fish intake in midlife are in line with a recent meta-analysis on 12 cohort studies [5], we emphasize that our study was conducted in a population with a uniquely high intake of particularly lean fish [26], [34], [35]. Hence, our reference group of up to two fish meals per week could in fact also be classified as a “high intake group” in other study populations. Thus, we cannot rule out the possibility that a potential beneficial threshold level might already have been reached by our reference group. Furthermore, it has been proposed that different methods of cooking fish might affect the prostate cancer risk where it is suggested that avoiding high-temperature cooking methods for white fish might lower the risk [13]. Although we do not have information on cooking methods in present study, information from a national nutrition survey conducted in 1990 showed that 64% of total fish consumed as a main meal was boiled or baked, while 36% was fried [35].

To our knowledge only one other study (population-based case-control) assessed early life fish intake and prostate cancer risk, and found a marginally increased risk following frequent fish consumption [21]. The discrepancy with our findings could be due to different study design, dissimilar fish species consumed, and different methods of collecting data on diet. We have previously reported that residency in seaside villages, with exceptionally high fish consumption for the first twenty years of life, was not associated with prostate cancer risk [19].

Unexpectedly, we discovered a positive association between frequent salted or smoked fish consumption both in early- and late life and advanced prostate cancer. At least three case-control studies have reported findings on this subject. A study from China assessed intake five years prior to diagnosis and reported positive association between salted fish consumption and total prostate cancer [36]. However a study from Poland [37] showed an inverse association between frequent consumption of smoked or dried fish or liver and a study from Canada also found an inverse association between frequent consumption of smoked/dried/salted fish and prostate cancer [38]. These mixed results could be due to different species of fish being preserved along with different methods of preserving the fish. In addition none of these studies presented data on advanced and localized prostate cancer separately. The mechanism behind our finding on salted or smoked fish is unclear and could be due to the salt content and/or presence of mutagens as a result of the preservation process [39]. Salt induces muscle lipid oxidation in fish [40] and lipid oxidation in n-3 or n-6 fatty acids generates α, β -unsaturated aldehydes supporting different functional groups containing oxygen, which are currently being considered as possible causal agents of different types of cancer [41]. Our findings could also be due to ineffective preservation processes ensuing in infectious microorganisms being present in the fish; genitourinary infection has been suggested to play a role in the etiology of prostate cancer, although specific infectious agent has yet to be identified [42].

We are not aware of studies that have examined smoked or salted fish consumption in early life in relation to prostate cancer, yet early life rural residency in Iceland (compared with early life city residency) examined in a larger cohort during the beginning and mid of 20^th^ century was associated with increased risk of advanced prostate cancer [19]. At that time high intake of milk, salted or smoked fish, meat and rye bread was typical. Thus, although we previously suggested that high milk consumption could explain our findings for rural residency, we cannot rule out that salted or smoked fish intake might explain, in part, the positive association between early life rural residencies and advanced prostate cancer risk.

We found no association between fish oil consumption in early-and midlife and prostate cancer, but late life consumption was inversely associated with advanced prostate cancer risk. This finding suggests a role in disease progression rather than etiology and fits with results on high prediagnostic plasma 25-hydroxyvitamin D predicting improved prostate cancer prognosis [17], [18].

The ability to study fish and fish oil consumption across the life course is a particular strength of our study design. Other important strengths are the extensive background data allowing control for potential confounding factors and the complete follow-up. For analyses that include prevalent cases, our results are vulnerable to recall bias because men with prevalent prostate cancer may evaluate their past dietary consumption differently from men without prostate cancer. However, for salted or smoked fish consumption, we only found associations with advanced prostate cancer not total, and only for early life intake, a pattern of findings unlikely to arise due to different recall between cases and controls. Furthermore, findings on current diet in late life were based on incident cases only.

The validation study on current food consumption in the AGES-Reykjavik did not show acceptable results for fish meals, possibly due to the inability of the 3-day food record used as a reference method, to adequately reflect individual intake of food items that are consumed 1–2 times per week or 3–4 times per week [28]. The validation study on midlife food consumption in the AGES-Reykjavik showed that participants were acceptably ranked by their intake of several important food groups [23]. Still, there is uncertainty in assessing dietary habits stretching over a 40 to 50 year period of time but this would typically lead to underestimation of the observed associations and failure to observe true associations. The validity of the early life dietary assessment has not and cannot be investigated. Yet, the data importantly show similar residency-dependent variation in dietary habits as documented in a household study conducted in Iceland in 1939 [25]. It has indeed been reported that food related memory from childhood over four decades later can be as accurate as from current diet, especially for food items eaten rarely or daily [43]. Another limitation to our study is the lack of information about total energy intake and fat intake; however we adjusted for body mass index measured in midlife, which may give indirect indication of total energy- and fat intake. Furthermore, we adjust for adult height, which can reflect nutritional status in early life [44]. Lastly, the frequency of fish oil consumption was not assessed in greater detail beyond “daily intake” which limited our opportunities for assessing dose-response. Daily dosage is however recommended on the product, which is 10 ml per day.

In summary, salted or smoked fish may increase risk of advanced prostate cancer, whereas in a setting with very high fish consumption no association was found between overall lean fish consumption in early life or midlife and prostate cancer risk. Potential exposure to carcinogens in salted or smoked fish needs further study. We observed reduced risk associated with fish oil consumption in late life, but not in early life or midlife, which may be an indication of a mechanism involving n-3 PUFAs and/or vitamin D and opens for studies on the potential protection of fish oil on the progression of prostate cancer. Improved understanding of potential dietary factors affecting prostate cancer risk, especially for advanced disease, could have a major public health impact.
